# Mechanical property analysis and dry sand three-body abrasive wear behaviour of AZ31/ZrO_2_ composites produced by stir casting

**DOI:** 10.1038/s41598-024-52100-9

**Published:** 2024-01-17

**Authors:** T. Satish Kumar, R. Raghu, Titus Thankachan, Robert Čep, Kanak Kalita

**Affiliations:** 1 Department of Mechanical Engineering, Amrita School of Engineering, Amrita Vishwa Vidyapeetham, Coimbatore, 641112 India; 2 Department of Mechanical Engineering, Sri Ramakrishna Engineering College, Coimbatore, Tamil Nadu India; 3 Department of Mechanical Engineering, Karpagam College of Engineering, Coimbatore, India; 4 Department of Machining, Assembly and Engineering Metrology, Faculty of Mechanical Engineering, VSB-Technical University of Ostrava, 70800 Ostrava, Czech Republic; 5 Department of Mechanical Engineering, Vel Tech Rangarajan Dr. Sagunthala R&D Institute of Science and Technology, Avadi, 600 062 India; 6 University Centre for Research and Development, Chandigarh University, Mohali, 140413 India

**Keywords:** Mechanical engineering, Materials science

## Abstract

An experimental study of three body abrasive wear behaviour of AZ31/15 vol.% Zirconium dioxide (ZrO_2_) reinforced composites prepared by stir casting has been carried out. Microstructural analysis of the developed composites was carried out and found out that the microstructure of the composites revealed a uniform distribution of ZrO_2_ particles with refinement in the grain size of the matrix from 70 to 20 µm. The alterations in the microstructure led to an enhancement in both hardness (68–104 HV) and tensile strength (156–236 MPa) due to Orowan strengthening, quench hardening effect and better bonding. Response surface methodology was applied to formulate the three-body abrasive wear test characteristics such as load, speed, and time. Three body abrasive test results were utilized to generate surface graphs for different combinations of wear test parameters revealed an increase in specific wear rate. The specific wear rate was observed to increase with increase in speed up to a certain level and then started to decrease. The lowest possible specific wear rate was obtained for an optimized load of 20 N and a speed of 190 ms^−1^. Scanning electron microscopic examination of wear-tested samples showed higher specific wear rate at higher loads with predominantly abrasion type material removal. In conclusion, this study makes a substantial contribution to the field by elucidating the complex relationships among microstructure, mechanical properties, and the three-body abrasive wear behavior of AZ31/ZrO_2_ composites. The determination of optimal wear conditions and the insights gained into wear mechanisms provide valuable information for designing materials, implementing engineering solutions, and advancing the creation of wear-resistant components across a range of industries.

## Introduction

As a result of the high strength-to-weight ratio, easy availability, remarkable machinability, and superior castability, magnesium alloys are ideally suited for automotive and aerospace applications^1,2^. Magnesium alloys, despite their appealing features have restricted applications because of their low strength, poor ductility, and poor cold workability^3–5^. The incorporation of nano-reinforcements has been shown to significantly alter these properties, contributing to the expansion of magnesium alloys' application scope^6^. Addition of micro- and nano-sized reinforcement to the matrix improves the mechanical properties of magnesium alloys synthesized by different processing methods such as spray forming, pressure infiltration, stir casting, and powder metallurgy. Stir casting is the most well-known of the currently accessible technologies due to its versatility and cost-effectiveness for bulk manufacture of near-net-shape parts^7^. However, composites prepared by this method find some limitations such as creation of porosity and non-isotropic characteristics due to the agglomeration of ceramic particles within the matrix^8–10^. These challenges have led to ongoing research into optimizing the stir casting process to minimize defects and enhance material properties^11,12^. Magnesium composites are used in the automotive sector for the wheels, rotors and suspension components and aircraft sector for wing spars, fuselage panels and engine components due to its low dense and high strength with better fatigue properties^13,14^. In medical sector, it is used for screws and plates in medical implants due to its biodegradability. Compared to other composites, a magnesium composite has lowest density, high strength to weight ratio, fatigue resistance, enhanced corrosion resistance and biodegradability^15–17^.

Using the stir-casting process, Rashad et al.^18^ developed a graphene nanoplatelet (GNP) reinforced AZ31 magnesium matrix composite. Microhardness and tensile properties of the composite samples were reported to be greater than those of the AZ31 alloy. Banerjee et al.^19^ synthesized tungsten carbide (WC) reinforced AZ31 composites using ultrasonication assisted stir casting. Addition of WC nanoparticles to the AZ31 alloy was reported to greatly increase the hardness, wear and tear, and friction of the composites. This is consistent with other studies that have found similar improvements in mechanical properties with the incorporation of various nano-sized reinforcements^20,21^. Nguyen et al.^22^ examined the wear characteristics of nano alumina (Al_2_O_3_) reinforced AZ31B composite fabricated by fragmented melt deposition. Under normal conditions, the wear rate of composites was observed to decline with sliding speed. The AZ91D/SiC particle-reinforced composite was synthesized using a two-step stir-casting method^23^. The results revealed an improvement in mechanical properties with addition of SiC. Fine SiC particles were reported to be more effective in enhancing the mechanical characteristics of composites in contrast with coarse SiC particles. The ductility of the matrix alloy was also reported to reduce with increase in the fraction of SiC particles in the matrix. Nagaraj et al.^24^ studied the wear parameters of AZX915 Mg-alloy composites reinforced with 12 wt% titanium carbide (TiC) particles. T4 thermal treatment was carried out to as-cast composites in an argon environment for 48 h at 420 °C. Wear studies revealed significant improvement in wear rate of the composites due to the incorporation of TiC particles. Zirconium dioxide (ZrO_2_) was chosen as the reinforcement owing to its high hardness, wear resistance and fracture toughness along with good oxidation resistance compared to other nanoparticles. Specifically, ZrO_2_ possess abrasion resistance which makes it suitable for this investigation to study it potential for three body abrasive wear applications.

According to a thorough review of the literature, there are no reports on the role of ZrO_2_ reinforcements on the wear behaviour of AZ31 alloy focusing the three body abrasive wear behaviour. As a result, the current work aims to analyse the three-body abrasive wear behaviour of the composite as a function of load, speed, and time by employing response surface method. The main idea/contribution of the paper is to investigate the three body abrasive wear behavior of the developed composite under different tribological conditions and finding of the optimum condition in which lowest abrasive wear rate is resulted. This insight will bring out the feasibility of the developed composite for its usage in tribological applications such as engine bearings, piston rings, piston, brake drum and cylinder liner^25,26^. To study the three-body abrasive wear behavior of the samples, analysis of variance (ANOVA) was performed. Regression analysis was used to build the model and to determine the relationship amid the parameters and the response. To further investigate the three-body wear mechanisms, the worn-out surface was analysed using Scanning electron microscopic (SEM).

## Experimental details

### Fabrication of AZ31/15 vol% ZrO_2_ composites

A composite material, AZ31/15 vol% ZrO_2_, was prepared using the stir casting technique. The AZ31 Mg alloy, serving as the matrix, consists of major elements with the following composition: 3.2 wt% Al, 0.9 wt% Zn, 0.3 wt% Mn, and 0.1 wt% Si. ZrO_2_ particles, with a particle size ranging from 1 to 5 µm, were used as the reinforcement. Initially, 400 g of the AZ31 alloy was melted at a temperature of 700 °C in a CaF_2_-SF_6_ shielding atmosphere. After reaching a molten state, 15 vol% of preheated ZrO_2_ particles (heated to 400 °C for 2 h) were added to the melt. To ensure a uniform dispersion of ZrO_2_ particles within the melt, the mixture was stirred for 10 min using a stainless steel stirrer at 600 rpm. The AZ31/ZrO_2_ mixture was then poured into a steel mold with dimensions of 150 mm × 50 mm × 60 mm and allowed to solidify. Subsequently, microstructural examination of the composite material was conducted using an optical microscope (MA-100, Nikon) to assess the distribution of ZrO_2_ reinforcement particles. Samples were prepared by polishing them according to standard metallographic procedures, and the polished specimens were etched using a solution composed of 4 ml picric acid, 10 ml acetic acid, and 70 ml ethanol. In addition to microstructural analysis, X-ray diffraction (XRD) studies were carried out using a Shimadzu X-ray diffractometer (XRD 6000) with Cu Kα (λ = 1.5409 Å) radiation. These analyses were likely performed to further characterize the composite's structural properties and identify the phases present. The Vickers microhardness testing of the composites was conducted in accordance with the ASTM E384 standard. A load of 100 g was applied for duration of 15 s during the testing process. To determine the tensile properties of the specimens, a tensile testing machine (specifically, an Instron 1195) was employed. The testing procedure followed the guidelines outlined in the ASTM E8/E8M-08 standards. To assess the strength of the specimen, tensile testing was carried out on the samples having wide of 6 mm and gauge length of 25.0 ± 0.1 mm at a strain rate of 0.006 s^−1^. The dry sand three body abrasive wear tests were carried out as per the ASTM G65 Standard. The dry abrasion wear tester has a chlorobutyl rubber wheel of 228 mm diameter that abrades the ZrO_2_ reinforced composite samples. The speed of the rotating wheel can be adjusted from 10 to 210 rpm. The composite samples are loaded in to the holder and load is applied over the specimen to keep it in contact with the rotating wheel. Silica sand with particle size ranging from 50 to 70 µm was made to fall through the hopper among the rotating composite specimen and rubber wheel at a controlled flow rate of 354 g/min. To perform abrasive wear test, specimens of 75 × 25 × 12.5 mm were cut from the cast of ZrO_2_ reinforced composite. The wear experiments were carried out at varying speeds, loads, and abrading times. Statistical studies were performed by means of MINITAB software and the Response surface methodology (RSM) for the different levels of parameters indicated in Table 1. The weight loss of the specimen was measured and the specific wear rate was measured using the given Eq. (1).

1 $${\text{Specific Wear rate }} = \frac{\Delta G}{{dMS}}$$

**Table 1:** Parameters and levels of wear testing.

Level	Load (N)	Speed (rpm)	Time (min)
1	20	70	2
2	40	130	6
3	60	190	10

Here, *∆G* is the weight loss of the specimen after wear test, *S* is the sliding distance, *M* is the load and *d* is the density of the composite.

### Response surface methodology

The Response Surface Methodology (RSM) integrates scientific and numerical approaches, employing regression analysis, statistical tools, and experimental design. Its primary goals encompass understanding how changes in input levels impact quality characteristics and identifying the ideal operating conditions or the range of factors meeting operational requirements. RSM excels in scenarios where multiple input variables influence the response. These controlled variables, termed factors, are managed by researchers. As the exact shape of the objective function remains unknown, RSM employs linear or quadratic polynomial equations to establish the relationship between factors (*X*_1_, *X*_2_, … *X*_k_) and the response (y). Typically, a second-order model is utilized in RSM.

2 $$y = \beta_{0} + \sum_{i = 1}^{k} \beta_{i} X_{i} + \sum \sum_{i < j} \beta_{ij} X_{i} X_{j} + \sum_{i = 1}^{k} \beta_{ij} X_{i}^{2} + \varepsilon$$

where *y* represents the predicted response, *I* signifies the linear coefficient, *j* stands for the quadratic coefficient, β represents the regression coefficient, *k* denotes the number of factors, and *ε* accounts for random experimental error assumed to have a zero mean.

Optimizing system performance hinges on identifying the most effective factor combinations. While the Taguchi method finds widespread use, its optimal solution often lies within one of the experiments. In contrast, RSM holds an advantage in determining optimal experiment levels through polynomial regression. The L20 matrix, part of fractional factorial designs in RSM, offers an efficient approach to explore numerous factors in a limited set of experimental runs. Its advantages stem from: By minimizing the number of experimental runs required, L20 designs still provide substantial data to accurately construct a response surface model. The selection of the L20 matrix involves weighing factors such as the number of factors to explore, desired resolution, and available resources. Researchers often favor this design due to its efficient exploration of a significant number of factors within a manageable number of experimental runs^27,28^.

The RSM method was used to design wear tests in order to reduce the number of trail experiments required to obtain optimum parametric parameters^29^. To optimise the response, statistical and mathematical techniques were employed to analyze the role of each input parametric condition on the outcome. Using central composite design (CCD)^30^, response surface design was applied with load, time, and speed as input parameters (with 3 operation levels as represented in Table 1). A sum of 20 trail experiments was produced and is given in Table 2.

**Table 2:** RSM design of experiments.

S. no	Load (N)	Speed (rpm)	Time (min)
1	60	70	2
2	40	130	6
3	40	130	6
4	40	130	2
5	60	190	2
6	40	130	6
7	20	190	10
8	60	190	10
9	40	130	6
10	20	130	6
11	40	130	6
12	40	130	6
13	40	190	6
14	60	130	6
15	60	70	10
16	20	190	2
17	20	70	2
18	40	70	6
19	20	70	10
20	40	130	10

## Results and discussion

### Microstructure of AZ31/ZrO_2_ MMCs

**Figure 1:**
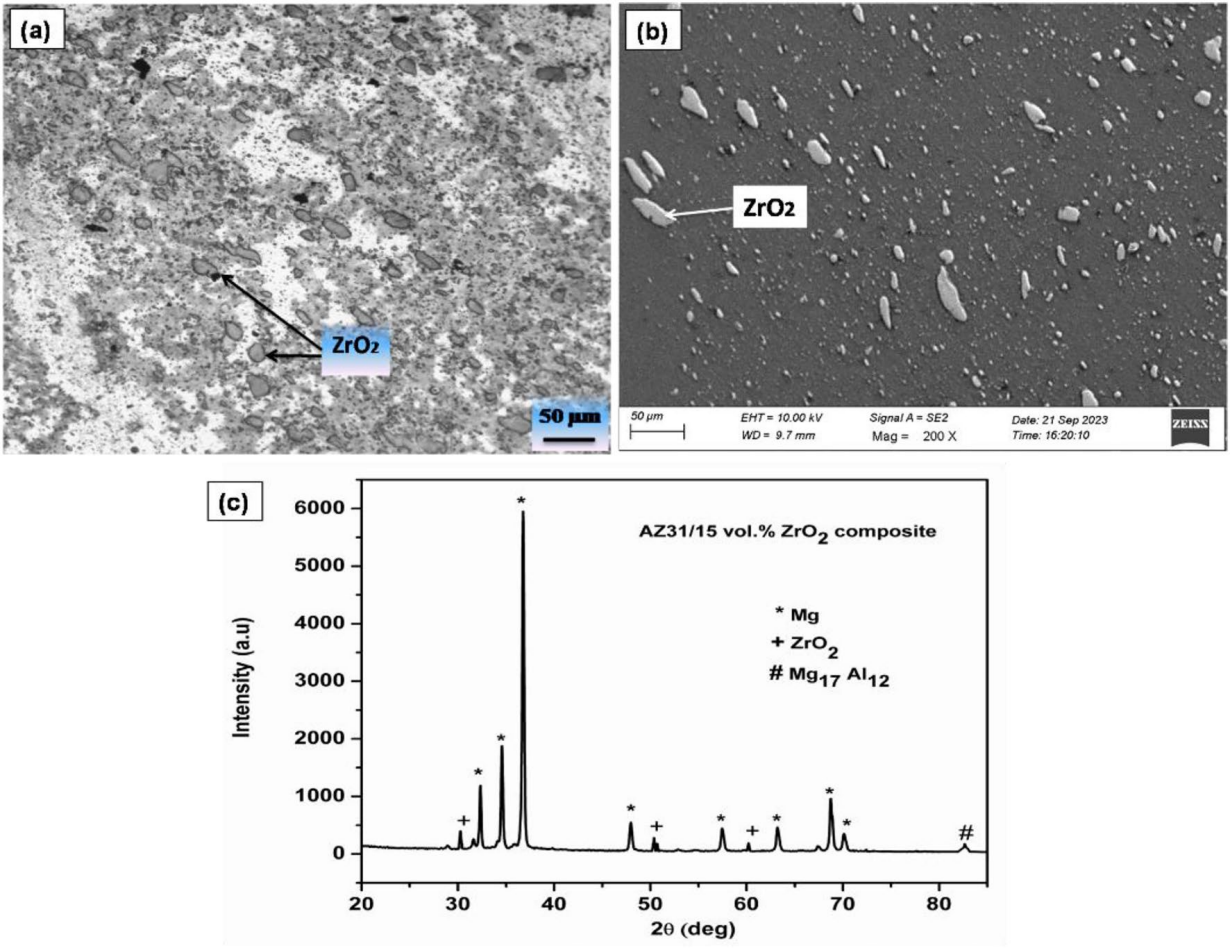
(**a**) Optical image, (**b**) SEM image and (**c**) XRD pattern of AZ31/15 vol% ZrO_2_ composite.

Figure 1a shows optical image and 1b shows SEM image of AZ31/ 15 vol% ZrO_2_ composite produced by stir casting technique. The X-ray diffraction (XRD) patterns in Fig. 1c illustrate the characteristics of AZ31/ 15 vol% ZrO_2_ composite. Within the XRD pattern, we can observe discernible peaks corresponding to magnesium, Mg_17_Al_12_ and ZrO_2_ particles. Notably, there are no discernible peaks corresponding to the any other compound or intermetallics. This observation suggests that the ZrO_2_ added has been completely stable and did not undergo transformation into other compounds through decomposition. The ZrO_2_ particles are found to be uniformly distributed in the matrix and are also found to significantly refine the grain size of the AZ31 matrix as shown in Fig. 1a. The 15 vol% ZrO_2_ particles added composites showed an average grain size of 20 µm, where as the AZ31 matrix as the initial grain size of about 70 µm as explained in the previous study^31^. Crystal grains form within the molten metal through a process involving nucleation and growth. The nucleation rate is affected by two primary factors: the free energy of the critical nucleus and the energy required for diffusion activation. Notably, as the high weight fraction increases, the nucleation rate also increases. This heightened nucleation rate may have consequences for the crystal's growth, potentially restricting it in one direction and leading to a change in its morphology.

### Mechanical properties of AZ31/ZrO_2_ MMCs

The incorporation of fine ZrO_2_ particles into the metallic matrix led to a notable enhancement in the microhardness of the composites. AZ31 alloy has a hardness of 68 HV, whereas AZ31/15 vol% ZrO_2_ added composite is found to exhibit a hardness of 104 HV. The presence of hard ZrO_2_ particles and fine grains effectively contributed to the improvement in hardness. The increase in microhardness can be attributed to several factors resulting from the reinforcement of ZrO_2_ particles, as described in previous studies^23,24,31,32^. First, the homogeneous distribution of FA particles activates Orowan strengthening. This uniform distribution creates resistance against the movement of dislocations, altering their path within the material. Furthermore, the thermal mismatch between ZrO_2_ particles and the metallic matrix generates varying degrees of dislocation density within the composites. This, in turn, results in a quench hardening effect, contributing to the observed increase in microhardness.

**Figure 2:**
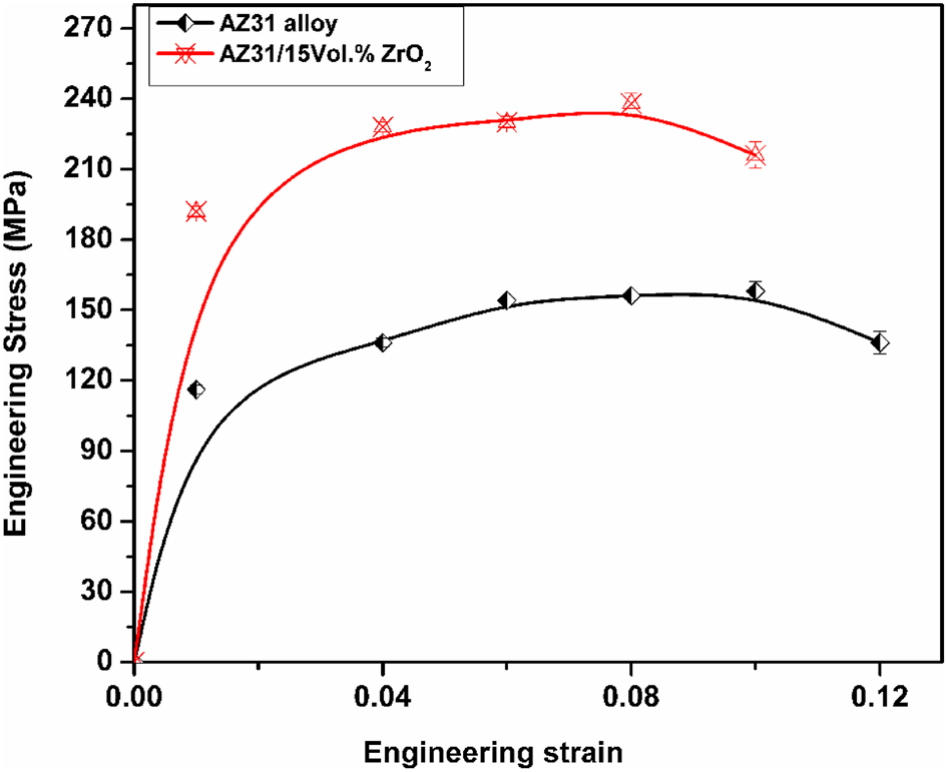
Tensile properties of AZ31/15 vol% ZrO_2_ composite.

In the study, the tensile behavior of AZ31/15 vol% ZrO_2_ composite was investigated. Figure 2 presents tensile properties comparison between the developed composites and the as-received AZ31 alloy. The ultimate tensile strength (UTS) of the matrix alloy was measured to be 156 MPa, while that of the composite was found to be 236 MPa (as shown in Fig. 2). This increase in strength can be featured to several factors, including grain refinement, consistent dispersion of particles, and the development of a robust interface between the matrix and the reinforcement. Effective load transfer from the matrix to the particles is facilitated by the strong interface present in the composite. This allows for the efficient utilization of the reinforcement during tensile loading.

### Abrasive wear behaviour

Using regression equations and surface plots, statistically abrasive wear tests and experimental results were analyzed by ANOVA to find the important parameter that influences the specific wear rate. The outcomes of the twenty experiments are provided in Table 3.

**Table 3:** Wear rate obtained from three body wear testing.

S. No	Load (N)	Speed (rpm)	Time (min)	Specific wear rate (mm^3^/Nm)	Friction coefficient
1	60	70	2	0.00072	0.58
2	40	130	6	0.00052	0.52
3	40	130	6	0.00051	0.52
4	40	130	2	0.00041	0.49
5	60	190	2	0.00062	0.56
6	40	130	6	0.00049	0.52
7	20	190	10	0.00028	0.42
8	60	190	10	0.00069	0.58
9	40	130	6	0.00049	0.52
10	20	130	6	0.00029	0.44
11	40	130	6	0.00049	0.52
12	40	130	6	0.00053	0.52
13	40	190	6	0.00039	0.48
14	60	130	6	0.00068	0.6
15	60	70	10	0.00079	0.59
16	20	190	2	0.00024	0.38
17	20	70	2	0.00026	0.35
18	40	70	6	0.00045	0.5
19	20	70	10	0.00027	0.4
20	40	130	10	0.00059	0.54

**Figure 3:**
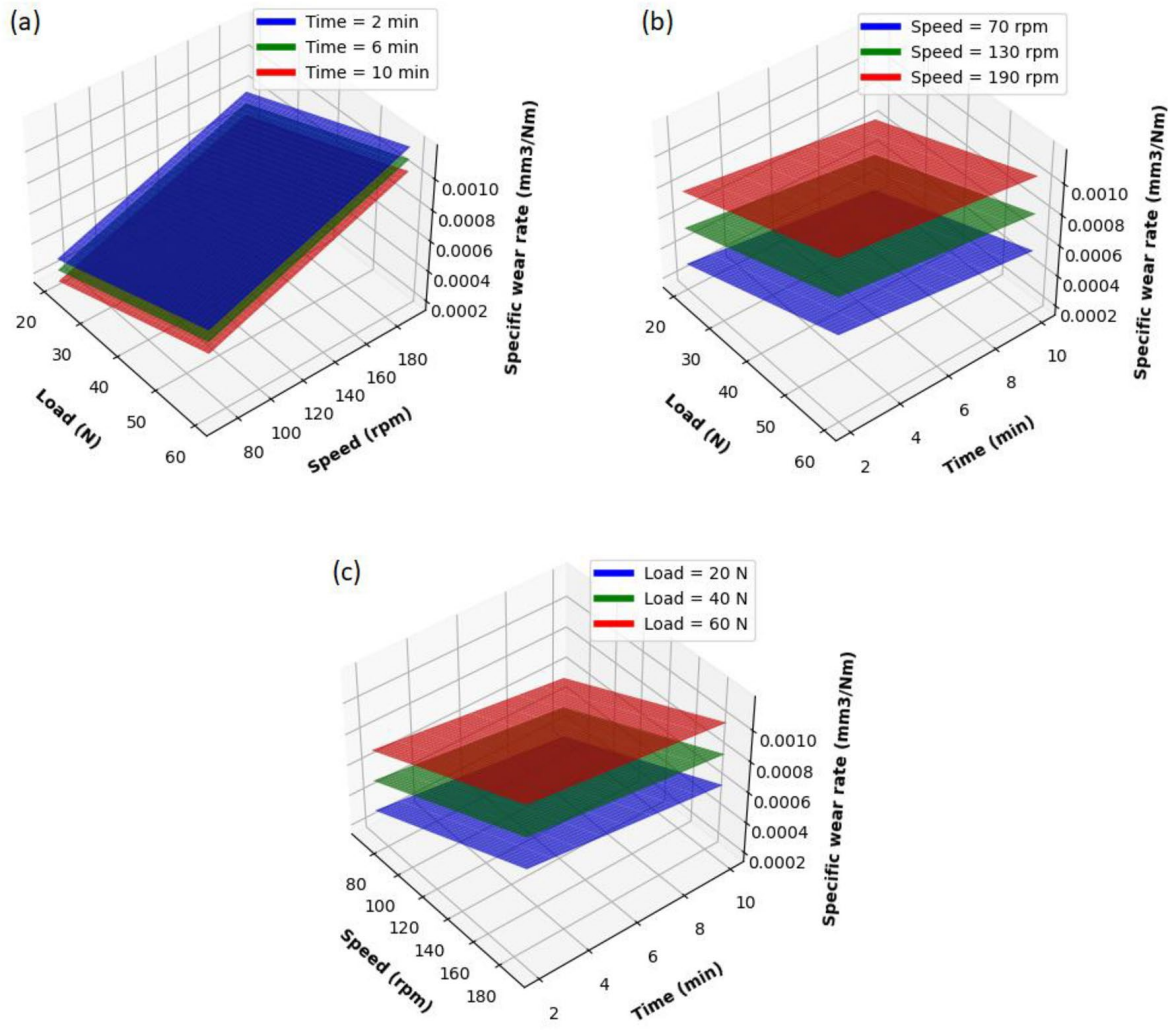
Surface plot of specific wear rate versus (**a**) load and speed, (**b**) load and time and (**c**) speed and time.

The specific wear rate acquired by conducting 20 experiments was consolidated and compared alongside all the input parameters, viz., speed, load, and time. 130 rpm speed, 40 N load, and 6 min time are the hold values set for the plots. The relationship between each parameter and response can be detailed with the aid of surface plots. The surface plots of specific wear rate verses (a) load versus speed, (b) load versus time duration, and (c) speed versus time duration are provided in Fig. 3. From Fig. 3a, b, it is understandable that specific wear rate increases as load increases. The pressure acting on the rubber wheel directly depends on the amount of load applied; as the load increases, the contact between the specimen and the wheel increases. Abrasion, cutting, and ploughing effects caused by the abrasive particles over the specimen depend on the applied load. The abrasive particles cutting action happens to be more severe at higher loads and thus leads to a high wear rate. Under high contact pressure, abrasive particles will penetrate the composite specimen, causing an increase in wear rate^33–37^. From Fig. 3a, c, it is noticed that wear rate increases at low speed initially and declines when reaching higher speed. This is because the specimen has minimal contact with the wheel at high speeds and may be due to the high surface temperature of the specimen at high speeds. From Fig. 3b, c it is noticed that it is clear that the sliding time did not significantly affect the wear rate. The wear rate got sliding increased with increase in time this is due to more interaction among the specimen and wheel, which leads to a higher wear rate.

### Mathematical model

The connection between the wear rate and the process parameters can be mathematically represented using regression Eq. (2), which is obtained by MINITAB. Confirmation experiments were performed for various time, load and speed to evaluate the generated mathematical model. The confirmation test results are given in Table 4. This confirms that the wear rates found in the regression equation and measured experimentally have very minimal differences. In all three cases, the error observed was not more than 6%. Therefore, it is found that the developed model (Eq. 3) correlates the process parameters and the response well adequately.

3 $$\begin{aligned} {\text{Specific wear rate }}\left( {{\text{mm}}^{{3}} /{\text{Nm}}} \right) = & - 0.000{131 } + \, 0.0000{1}0{\text{ Load }}\left( {\text{N}} \right) \, \\ & + \, 0.00000{\text{4 Speed }}\left( {{\text{rpm}}} \right) - \, 0.0000{\text{19 Time }}\left( {{\text{min}}} \right) \\ \end{aligned}$$

**Table 4:** Confirmation experiments and regression values with error calculation.

S. No	Load (N)	Speed (rpm)	Time (min)	Experimental wear rate	Regression wear rate	Error (%)
1	30	100	3	0.00054	0.00051	5.88
2	30	150	5	0.00069	0.00067	2.98
3	50	150	5	0.00089	0.00087	2.29

### Analysis of variance

The developed model accuracy was verified using an ANOVA tool. The abrasive wear results are analyzed by keeping a 95% confidence level and a 5% significance level. The lack-of-fit is 5.05 for the standard F value at confidence level of 95% for the model developed, which is 7.55 as shown in Table 5, which confirms that the developed model gives good results. The terms with P values less than 0.05 suggested that they had a substantial influence on the response. Load observed to the most influencing parameter (90.72%) on the specific wear rate followed by time (2.66%) and speed (1.42%). The interaction effect of the different combination of the parameters on the wear rate are not significant since the P value of the 2 way interaction of different combination of the parameters lies greater than the typical significance level of 0.05. This indicates that the interaction effect is not significantly different from zero. The majority of variation (as indicated by the Sum of Squares) comes from the Linear Model Term, suggesting that much of the variability in the dependent variable is explained by the factors included in the model, hence the model is well adequate. The R-sq and adj R-sq values are found to be 97.42% and 95.11% which are closer to each other indicating that the model developed through this analysis has adequacy.

**Table 5:** ANOVA for three body abrasive specific wear rate.

Source	*DF*	Specific wear rate					Friction coefficient				
		Seq SS	Contribution (%)	Adj SS	F-value	*P* value	Seq SS	Contribution (%)	Adj SS	F value	*P* value
Model	9.00	0.00^a^	97	0.00	42.03	0.00	0.10	99	0.0950	128.34	0.00
Linear	3.00	0.00	95	0.00	122.68	0.00	0.09	91	0.0875	354.63	0.00
Load	1.00	0.00	91	0.00	352.20	0.00	0.08	88	0.0846	1028.77	0.00
Speed	1.00	0.00	1	0.00	5.50	0.04	0.00	0	0.00	0.00	1.00
Time	1.00	0.00	3	0.00	10.33	0.01	0.00	3	0.0029	35.13	0.00
Square	3.00	0.00	2	0.00	1.98	0.18	0.01	7	0.0063	25.33	0.00
Load × load	1.00	0.00	0	0.00	0.31	0.59	0.00	3	0.00	0.03	0.87
Speed × speed	1.00	0.00	1	0.00	5.77	0.04	0.00	4	0.0026	31.93	0.00
Time × time	1.00	0.00	0	0.00	1.54	0.24	0.00	0	0.0001	1.17	0.31
2-Way interaction	3.00	0.00	1	0.00	1.42	0.29	0.00	1	0.0013	5.06	0.02
Load × speed	1.00	0.00	1	0.00	3.41	0.10	0.00	1	0.0008	9.72	0.01
Load × time	1.00	0.00	0	0.00	0.76	0.40	0.00	0	0.0005	5.47	0.04
Speed × time	1.00	0.00	0	0.00	0.08	0.78	0.00	0	0.00	0.00	1.00
Error	10.00	0.00	3	0.00			0.00	1	0.0008		
Lack-of-fit	5.00	0.00	2	0.00	7.55	0.02	0.00	1	0.0008		
Pure error	5.00	0.00	0	0.00			0.00	0	0.00		
Total	19.00	0.00	100				0.10	100			

^a^0.00 indicates values < 0.0001.

As likely in case of specific wear rate, load has exerted the most influence on the friction coefficient (88.30%), followed by time (30.01%). The majority of variation, as indicated by the Sum of Squares, stems from the Linear Model Term and Load, implying that the included factors explain much of the variability in the dependent variable. The R-squared and adjusted R-squared values, 99.14% and 98.37% respectively, closely align, indicating the adequacy of the model developed in this analysis as shown in Table 5.

### Optimization of specific wear rate

Responses were further optimised using the RSM technique to find the best parameter combination that gives the wear rate minimum. The main objective is to reduce the specific wear rate, and MINITAB response optimizers were used with maximum wear rate input. With a load of 20 N, 190 rpm, and 2 min, the findings exhibited a minimum wear rate of 0.00024 mm^3^/Nm.

### Scanning electron microscope study

**Figure 4:**
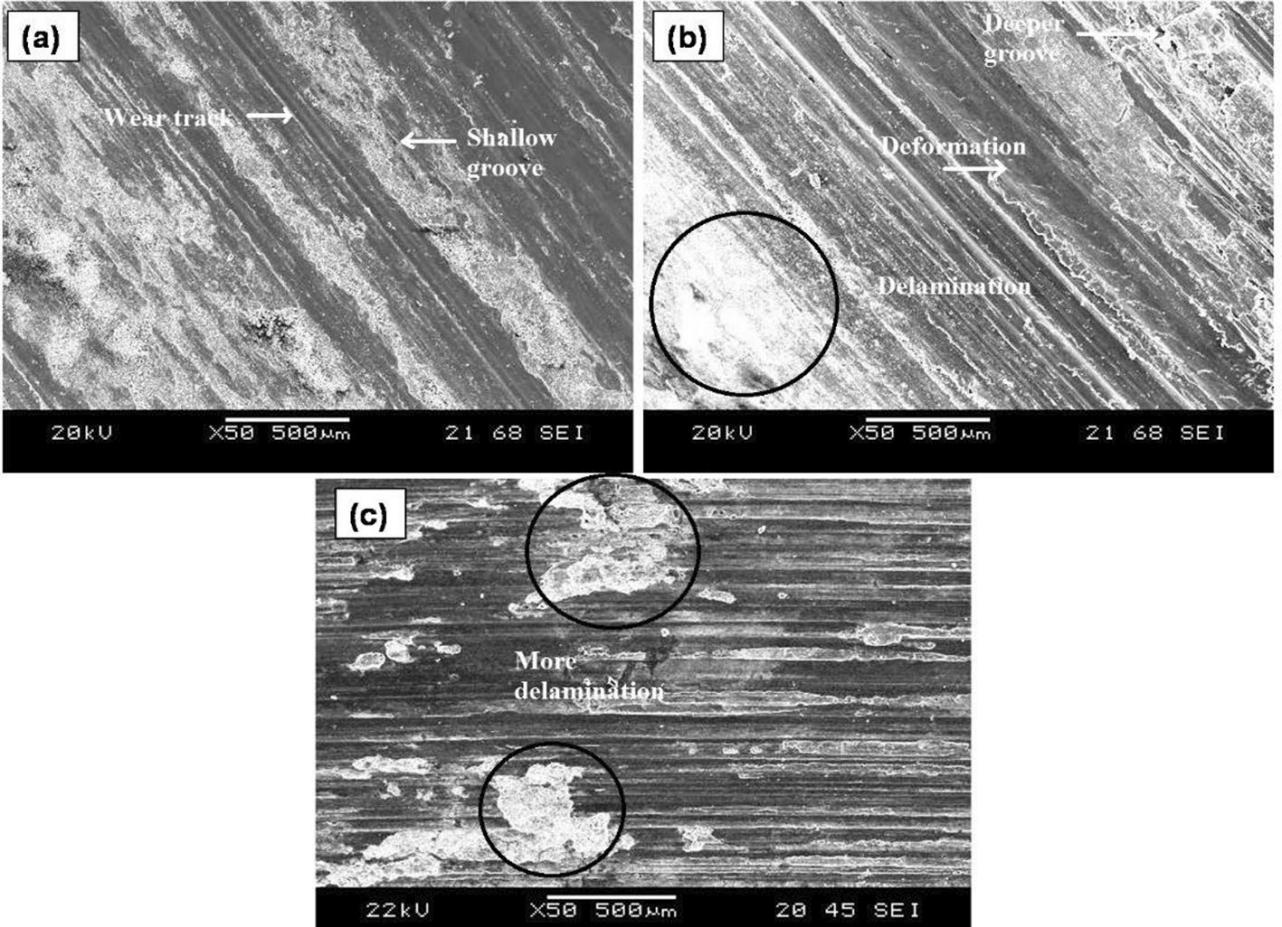
SEM images of AZ31/15 vol% ZrO_2_ composites were wear tested at applied loads of (**a**) 20 N, (**b**) 40 N and (**c**) 60 N with a common 6 min sliding time and a sliding speed of 130 rpm.

AZ31/15 vol% ZrO_2_ reinforced composites were wear tested at applied loads of 20, 40, and 60 N with a common sliding time of 6 min and a sliding speed of 130 rpm, examined using SEM. Samples worn tested at 20 N (Fig. 4a) revealed the presence of shallow grooves and fine scratches with very minimal material removal. This trend is mainly due to the minimum physical contact pressure between the rubber wheel and specimen. Less pressure at low loads leads to more rolling action of the abrasive particles, and ploughing action becomes ineffective. At an applied load of 40 N, the sample surface was subjected to more delamination, as illustrated in Fig. 4b. Abrasive particles will be subjected to crushing effects at a 40 N load, and hence more material removal was observed. With a further increase in load to 60 N, deeper grooves are clearly noticeable over the abraded specimen surface from Fig. 4c. Formations of deeper grooves are mainly due to the penetration of the abrasive particles; at higher loads, the abrasive particles will get compressed among the rubber wheel and specimen. At 60 N, severe delamination is clearly observed from the sample surface, which leads to high material removal^37^.

In the abrasive wear test, three distinct zones are identified: the entrance zone (where sand particles undergo crushing), the middle zone (experiencing maximum pressure at the interfaces), and the exit zone (where sand particles fall freely). The wear rate escalates with an increase in the load applied to the surface. During the experimental run, silica sand abrasive particles interact with both the specimen and the rubber wheel. These particles falling onto the specimen are pressed against it by the rubber wheel, simultaneously leading to sliding between the rubber wheel and the silica sand particles. The irregularly shaped silica sand abrasive particles cause cutting and ploughing actions on the specimen surface, with the extent of abrasion directly tied to the applied load. Under low load conditions, no trapping of the silica sand abrasive particles occurs, allowing for a free rolling effect between the specimen and the rubber wheel. Larger silica sand particles that come into contact with the specimen surface create mild abrasion. Notably, several indentation sites form on the specimen surfaces under low-load conditions. As the load increases, sand particles tend to be crushed in the entrance zone, intensifying the abrasive action on the specimen surface. The crushed particles, during wheel rotation, produce sliding actions, amplifying the cutting and ploughing effect on the specimen surface. Under high-load conditions, a larger surface area of the specimen comes into contact with both the rubber wheel and the silica sand particles. Consequently, these particles penetrate the specimen's surface due to increased pressure, resulting in higher material removal. However the order of the wear rate is of lower magnitude (× 10^–4^) even at higher load conditions which evident that incorporation of the ZrO_2_ in the AZ31 matrix offers greater abrasion resistance towards the abrasive action of the sand particles.

**Figure 5:**
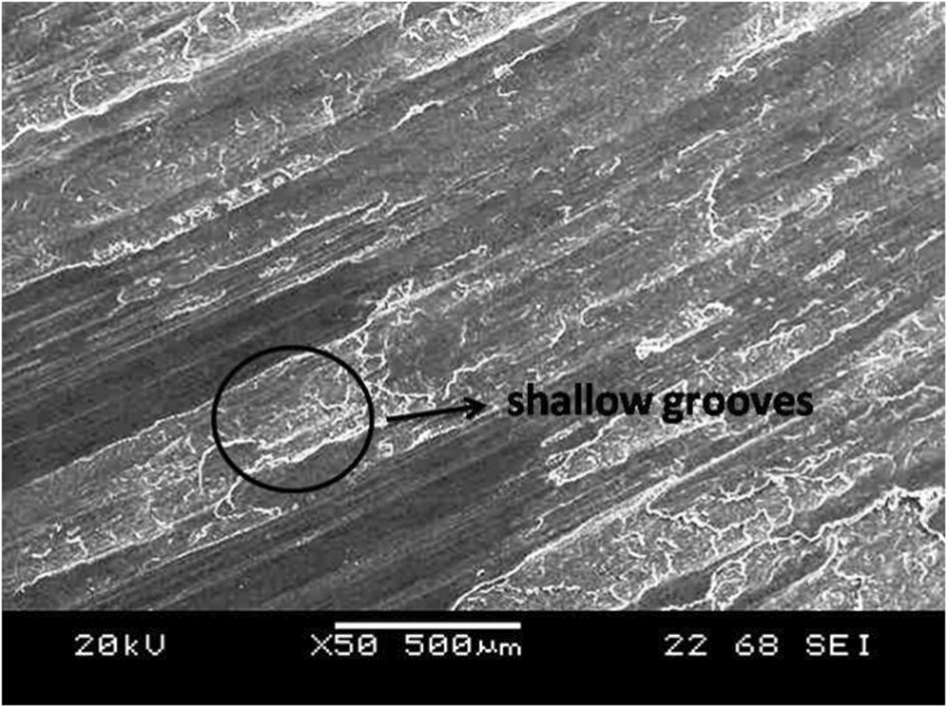
Shows the SEM image of the composites sample wear tested in the optimum conditions of 190 rpm speed, 20 N load and 2 min time.

Furthermore, the rubber wheel gets implanted abrasive particles at higher loads, and composite material removal increases due to the increased abrasion origin of the implanted abrasive particles. Figure 5 reveals the SEM micrograph of the composites sample wear tested in the optimum conditions of 190 rpm speed, 20 N load and 2 min time. It shows shallow grooves with minimum wear loss.

The wear loss of the specimen through different wear mechanisms is predominantly influenced by the hardness offered by the ZrO_2_ particles in the composite. Typically, abrasive particles that come between the specimen and the rubber wheel cause abrasion on the specimen's surface. The reinforcement in the surface acts as a harder layer covering the softer aluminum matrix. When silica sand abrasive particles come into contact with this robust reinforcement region, they are deflected into the rotating rubber wheel. This deflection occurs due to the resistance offered by the hard region against the destructive action of the silica sand particles. Essentially, the hard region protects the softer region from abrasion until it receives support from the softer region. This resistance reduces the cutting and ploughing action of the silica sand abrasive particles on the specimen surface, resulting in a lower order of wear rate. Therefore, based on the observed lower wear rate, it can be inferred that effective matrix support for the reinforcement particles and minimal protrusion of the reinforcement particles on the surface contribute to greater wear resistance. Despite the observed strong bonding at the interface between the hard reinforcement and the matrix, these interfaces are prone to micro-crack formation. Micro cracks initiate at various points along the matrix/reinforcement interface and propagate individually in different directions, eventually merging into larger cracks. This process leads to material removal from the specimen surface. However, the strength and hardness of the reinforcement particles counteract the negative impact of micro-cracking tendencies, enhancing the wear resistance of the developed composite to a significant extent.

This study provides in-depth insights into the changes in microstructure and improvements in mechanical properties. It introduces a robust model for predicting wear rates across varied conditions, enhancing the understanding of wear behavior in AZ31/ZrO_2_ metal matrix composites. The identification of optimal parameters for minimizing wear rates underscores the novelty and immediate practical relevance of the research. Moreover, the investigation into dry sand three-body abrasive wear in the developed composite highlights its potential for critical applications. Specifically, these composite exhibits promise in components such as brake/rotating discs, pistons, and liners, where the imperative characteristics of high wear resistance and low coefficient of friction (COF) are crucial for optimal performance.

While this study has made substantial contributions by uncovering insights into microstructural changes, improvements in mechanical properties, and the wear behavior of AZ31/ZrO_2_ metal matrix composites, it is essential to acknowledge certain limitations. Firstly, it's worth noting that the wear tests were conducted under specific conditions, and the findings may not fully represent the composite's performance in diverse operational environments. Future research could explore a broader range of wear conditions to provide a more comprehensive understanding of the material's behavior. Secondly, the study primarily focused on three-body abrasive wear, leaving other wear mechanisms relatively unexplored. Examining additional wear types, such as two-body abrasive wear or erosive wear, would contribute to a more thorough assessment of the composite's overall wear resistance. Furthermore, while the wear rate prediction model developed in this study is reliable within the tested parameters, additional validation under different environmental and load conditions would enhance its robustness. Extending the model's applicability to a wider range of scenarios could improve its predictive capabilities. For future research endeavors, it would be valuable to delve into the long-term durability and stability of the AZ31/ZrO_2_ composite in practical applications. Real-world conditions, such as variations in temperature, humidity, and cyclic loading, could be considered to simulate more realistic scenarios. In summary, while this study significantly advances our understanding of the AZ31/ZrO_2_ composite's wear behavior, acknowledging these limitations and exploring the suggested avenues for future research would contribute to a more nuanced and comprehensive body of knowledge in the field.

## Conclusion

The stir-casting method was used to produce AZ31/ZrO_2_ MMC. The composite specimens' microstructures exhibited a homogeneous dispersion of ZrO_2_ particles. The addition of ZrO_2_ particles to the AZ31 alloy resulted in an enhancement of mechanical properties. Specifically, the microhardness increased from 68 Hv at 0 vol% to 104 Hv at 15 vol% of ZrO_2_. Additionally, the ultimate tensile strength (UTS) improved from 156 MPa at 0 vol% to 236 MPa at 15 vol% of ZrO_2_.The composite wear properties were examined using a three-body abrasive wear test, and the wear rates obtained were plotted alongside the wear parameters. The rate of degradation increased with load and time. The highest wear rate is 0.00079 mm^3^/Nm, while the smallest is 0.00024 mm^3^/Nm. A regression equation was also developed, and validation trials demonstrated that the model was reliable, with a maximum error of 5.88%. ANOVA was used, and the model's sufficiency was proven by its low F value (7.55). Optimizing the wear rate resulted in the lowest wear rate of 0.00024 mm^3^/Nm for 190 rpm, 20 N load, and 2 min time. The samples were subjected to SEM investigation, which revealed that as the load increased, so did the rate of material removal thereby increasing the wear rate. The examination of dry sand three body abrasive wear in the developed composite has played a crucial role in recognizing its potential applications. This composite shows promise for use in components such as brake/rotating discs, pistons, and liners, addressing the essential wear-related requirements of high wear resistance and low coefficient of friction (COF).

## Data Availability

The data presented in this study are available through email upon request to the corresponding author.
